# In Vivo Imaging of Intraocular Fluidics in Vitrectomized Swine Eyes Using a Digital Fluoroscopy System

**DOI:** 10.1155/2016/9695165

**Published:** 2016-04-03

**Authors:** Tamer Tandogan, Ramin Khoramnia, Gerd Uwe Auffarth, Michael Janusz Koss, Chul Young Choi

**Affiliations:** ^1^David J Apple International Laboratory for Ocular Pathology and International Vision Correction Research Centre (IVCRC), Department of Ophthalmology, University of Heidelberg, Im Neuenheimer Feld 400, 69120 Heidelberg, Germany; ^2^Department of Ophthalmology, Kangbuk Samsung Hospital, Sungkyunkwan University School of Medicine, 29 Saemunan-ro, Jongno-gu, Seoul 03181, Republic of Korea

## Abstract

*Purpose*. To describe the characteristics of intraocular fluidics during cataract surgery in swine eyes with prior vitrectomy.* Methods*. We prepared three groups of enucleated swine eyes (nonvitrectomized, core, and totally vitrectomized). Irrigation and aspiration were performed (2.7 mm conventional sleeved phacosystem) using a balanced saline solution mixed with a water-soluble radiopaque contrast medium at 1 : 1 ratio. We imaged the eyes using a digital fluoroscopy system (DFS) during phacoemulsification and compared the characteristics of the intraocular fluid dynamics between the groups.* Results*. The anterior chamber depth (ACD) after the commencement of irrigation differed between groups (2.25 ± 0.06 mm; 2.33 ± 0.06 mm; 3.17 ± 0.11 mm), as well as the height of the fluid flowing from the anterior chamber into the posterior cavity that was identified by lifting up the iris to correct the infusion deviation syndrome (0.00 ± 0.00 mm; 0.41 ± 0.04 mm; 2.19 ± 0.35 mm).* Conclusions*. DFS demonstrated differences in fluid dynamics during phacoemulsification in swine eyes with or without prior vitrectomy. In completely vitrectomized eyes, the large ACD, which developed during phacoemulsification, could be reduced by lifting the iris and allowing the fluid to shift to the posterior cavity. Recognizing the differences in fluidics of vitrectomized eyes as compared to those of the nonvitrectomized eyes may reduce the frequency of intraoperative complications.

## 1. Introduction

With progressive refinements in vitreoretinal surgical techniques, an increasing number of posterior segment disorders are being successfully managed with pars plana vitrectomy. However, cataract formation is a frequent complication after vitrectomy, occurring in up to 80% of cases. Well-known potential complications that may arise from cataract surgery after vitrectomy include poor pupil dilation, posterior synechiae, zonular damage, posterior capsular tears, increased mobility of the iris-lens diaphragm, and altered intraocular fluid dynamics as a result of the absence of the anterior hyaloid surface [1]. Upon first entry of the phacotip into the anterior chamber, characteristically, the iris-lens diaphragm bows posteriorly as soon as irrigation begins, causing the anterior chamber to deepen excessively and the pupil to dilate widely. In 2003, Ahfat et al. described this phenomenon as infusion deviation syndrome (IDS): the initial deepening of the anterior chamber followed by a sudden and unpredictable shallowing [2]. The most important feature of these eyes is the lack of vitreous support. A phakic, vitrectomized eye has a posterior segment filled with fluid that lacks the properties of a gel due to the absence of collagen and hyaluronic acid [3].

Among studies on intraocular fluid dynamics, one particular study used a model that evaluated the fluidics of the anterior chamber using microparticles. The anterior flow could be documented with the help of a camera and a computer that monitored the flow of the microparticles [4].

Fluoroscopy is an imaging technique commonly used by physicians to obtain real-time images of the internal structures of a patient through the use of a fluoroscope such as for dynamic digital cardiovascular imaging applications.

Modern fluoroscopes couple the screen to an X-ray image intensifier and charge-coupled device (CCD) video camera, allowing the images to be recorded and played on a monitor.

The purpose of this study was to investigate the exact mechanism of IDS using a digital fluoroscopy system (DFS) in real time. We also sought to compare the changes in the iris, lens, and anterior hyaloid surface according to the degree of vitrectomy and to compare the intraocular fluid dynamics according to these changes.

## 2. Methods

**Figure 1:**
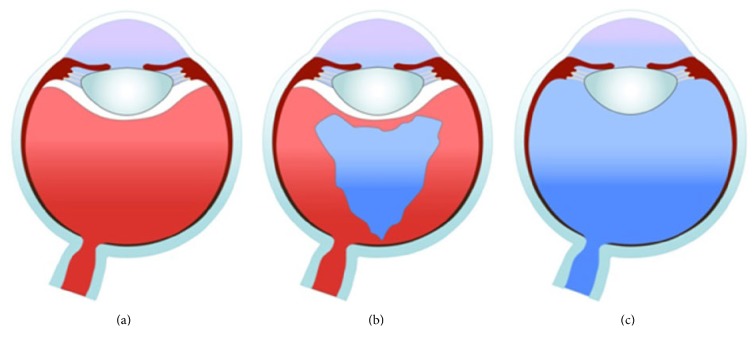
(a) Nonvitrectomized, (b) core vitrectomized, and (c) totally vitrectomized swine eyes.

This study was performed on enucleated eyes of pigs which were 5–8 months old having an axial length of around 24 mm. They were all studied within six hours after enucleation. Nine swine eyes were divided into three groups of three eyes each to compare the intraocular fluid dynamics according to the degree of vitrectomy. Groups 1, 2, and 3 were classified as normal swine eyes that did not undergo vitrectomy (Figure 1(a)), those that underwent core vitrectomy (Figure 1(b)), and those that underwent total vitrectomy (Figure 1(c)), respectively. The eyes of Groups 2 and 3 underwent a pars plana, 23-gauge, 3-port vitrectomy in the usual manner.

Images were taken using iodixanol (Visipaque, GE Healthcare, 320 mg-I/mL, osmolality 290 mOsm/kg water), which is a nonionic, isoosmolar contrast medium (Table 1). The contrast medium was mixed with a balanced saline solution (BSS) in a 1 : 1 ratio and then heated up to 37°C just before the experiment [5].

In each swine eye, the anterior chamber was irrigated with contrast medium mixed with BSS through a clear corneal incision (2.75 mm), using the Infiniti Phaco System (Alcon Laboratories), with the same parameters (aspiration flow rate: 30 mL/min, bottle height: 76 cm) for each procedure. We used a 30-degree phacotip with a 0.9 mm outer diameter.

In each group, the initial change of the anterior chamber depth (ACD) after irrigation with contrast medium was observed. If present, the degree of IDS was measured and the iris was lifted with a second instrument for 10 seconds. We observed the flow of irrigation fluid from the anterior chamber to the posterior cavity and recorded the amount of contrast medium in the posterior cavity.

**Figure 2:**
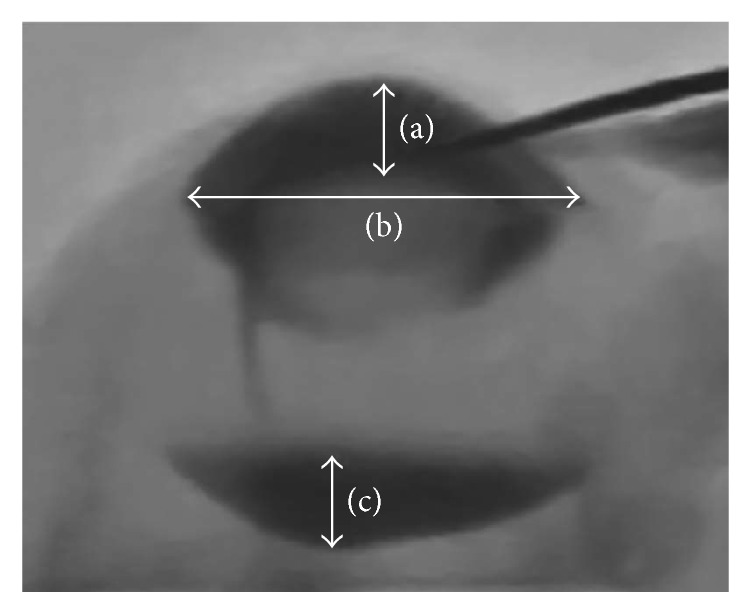
Fluoroscopic image of a swine eye: (a) anterior chamber depth, (b) angle-to-angle length, and (c) height of the contrast medium in the posterior cavity.

The flow of the contrast medium in the eyes was filmed during phacoemulsification using the DFS (AXIOM Artis dFC, Siemens, USA) at a speed of 30 frames per second. The whole imaging system enables a very high resolution of 184 *μ*m pixel pitch (956 × 954 pixels for a 9.76-inch diagonal). We compared the changes of the ACD and the amount of contrast medium in the posterior cavity before and after lifting the iris with a second instrument using still images taken from the real-time DFS. In this study, ACD was measured as the distance from the posterior corneal surface to the anterior lens surface, and the height of the contrast medium in the posterior cavity was measured as the distance from the anterior surface of the contrast medium to the anterior retinal surface (Figure 2). To compensate for the variation in image size, the angle-to-angle length of each eye was standardized to 10.0 mm. In each eye, the ACD and height of the contrast medium in the posterior cavity were also normalized using the same conversion ratio of angle-angle-length, and we compared the ACD and height of the contrast medium in the posterior cavity. Comparisons were made using the paired *t*-test. A *P* value of < 0.05 was considered to be statistically significant.

## 3. Results

**Figure 3:**
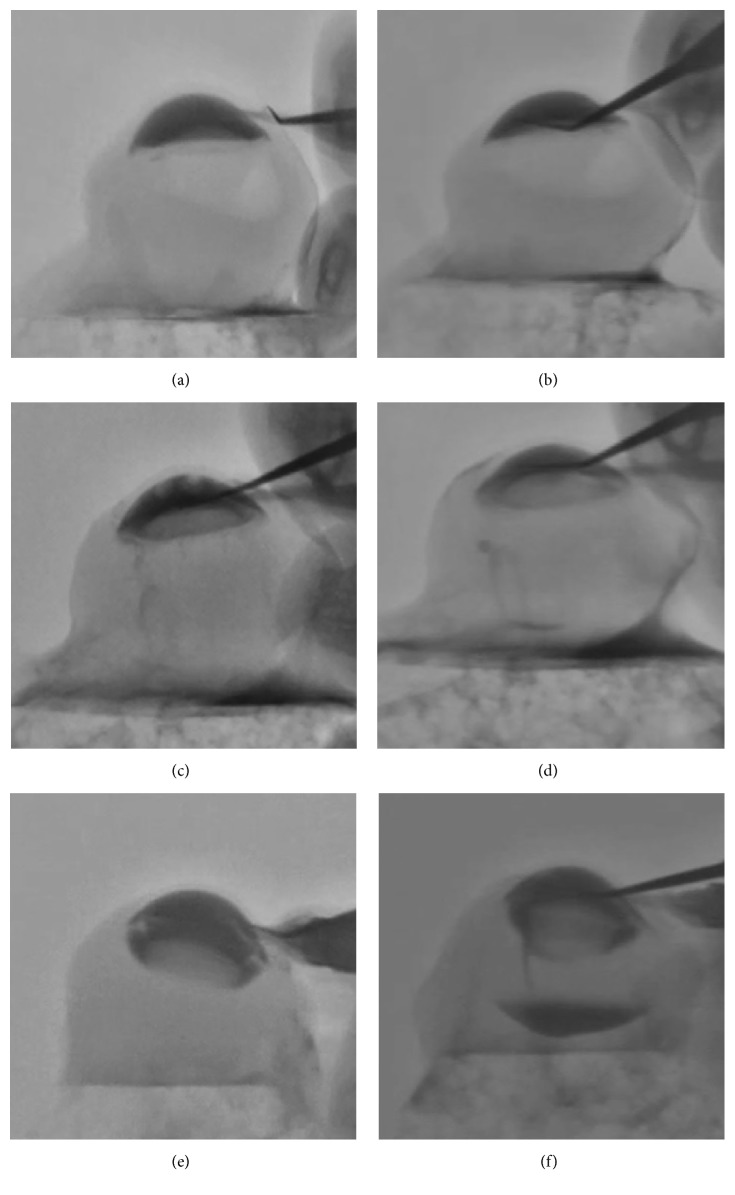
Fluoroscopic images of a swine eye (a, c, e) before lifting the iris and (b, d, f) after lifting the iris; (a, b) nonvitrectomized, (c, d) core vitrectomized, and (e, f) totally vitrectomized swine eyes.

In the nonvitrectomized eyes (Figure 3(a)), the iris-lens diaphragm did not bow posteriorly after irrigation began (mean ACD: 2.25 ± 0.06 mm). When the iris was lifted with the second instrument (mean ACD: 2.22 ± 0.05 mm), irrigation fluid did not pass into the posterior cavity (Figure 3(b)). In the core vitrectomized eye (Figure 3(c)), limited posterior bowing of the iris-lens diaphragm occurred (mean ACD: 2.33 ± 0.06 mm). After lifting the iris with the second instrument (mean ACD: 2.28 ± 0.07 mm), a small amount of irrigation fluid passed into the posterior cavity (mean height of the contrast medium in the posterior cavity: 0.41 ± 0.04 mm, (Figure 3(d))). We could also observe the narrow space between the posterior lens capsule and the relatively intact anterior hyaloid surface, and, in some areas, the real-time fluid shifts from the anterior chamber to the posterior cavity with the DFS. However, in the totally vitrectomized eyes, the iris-lens diaphragm bowed markedly as soon as the irrigation began (mean ACD: 3.17 ± 0.11 mm) (Figure 3(e)). When the iris was lifted with the second instrument, a large amount of fluid passed into the posterior cavity (mean height of the contrast medium in the posterior cavity: 2.19 ± 0.35 mm), and then the ACD was normalized (Figure 3(f), mean ACD: 2.32 ± 0.03 mm).  Figure 3 shows the different chamber depths before the iris was lifted and amounts of dye that passed from the anterior chamber into the posterior cavity after lifting the iris.

**Figure 4:**
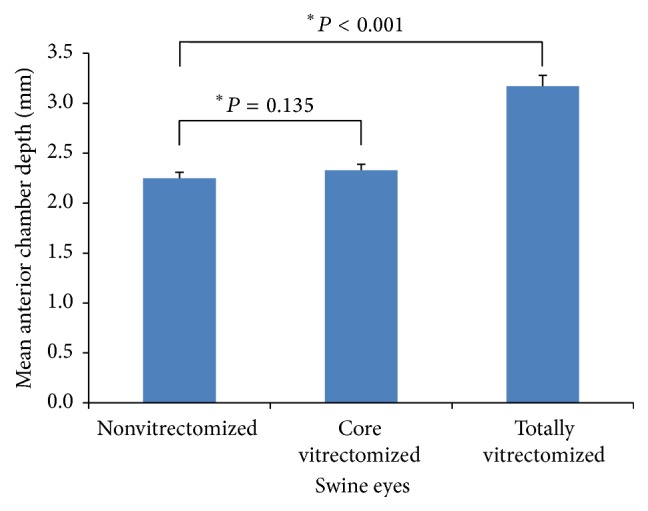
Mean anterior chamber depth after commencement of irrigation.

**Figure 5:**
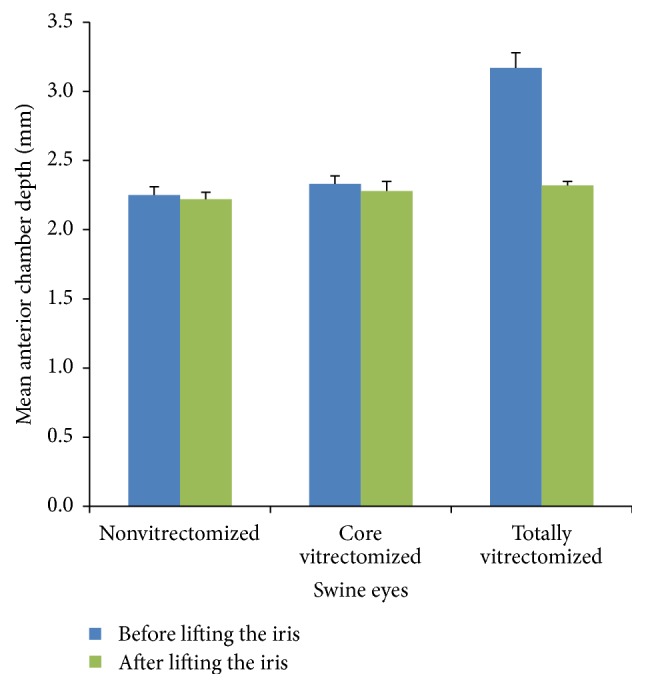
Change of mean anterior chamber depth before and after lifting the iris. In the nonvitrectomized eyes, the mean change of the ACD was not significant (*P* = 0.094). However, in the core vitrectomized and totally vitrectomized swine eyes, the mean changes of the ACD were significant (*P* = 0.023, *P* = 0.009, resp.).

**Figure 6:**
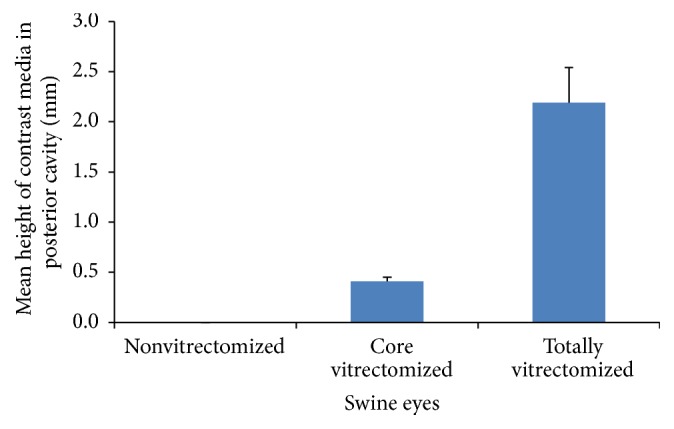
Mean height of the contrast medium in the posterior cavity of the eyes according to group. The mean height of the contrast medium in the posterior cavity in the totally vitrectomized eyes was significantly greater than that of the core vitrectomized eyes (*P* = 0.001). However, there was no flow of contrast medium into the posterior cavity in the nonvitrectomized eyes.

After irrigation commenced, the mean ACD of the core vitrectomized eyes was not significantly deeper than that of the nonvitrectomized eyes (*P* = 0.135). However, the mean ACD of the totally vitrectomized eyes was significantly deeper than that of nonvitrectomized eyes (*P* < 0.001, (Figure 4)). In the case of the nonvitrectomized eyes, the change in the mean ACD after lifting the iris was not significant (*P* = 0.094). However, in the core vitrectomized eyes, the change in the mean ACD was significant (*P* = 0.023), as well as in the totally vitrectomized eyes (*P* = 0.009 (Figure 5)). The mean height of the contrast medium in the posterior cavity of the totally vitrectomized eyes was significantly greater than that of the core vitrectomized eyes (*P* = 0.001). There was no contrast medium in the posterior cavity of the nonvitrectomized eyes (Figure 6).

## 4. Discussion

Infusion deviation syndrome (IDS) is caused by a lack of vitreous support and posterior bowing of the iris-lens diaphragm. IDS can result in the blockage of fluid passage from the anterior chamber to the vitreous cavity, causing significant pressure differences between the anterior and posterior compartments [3]. This phenomenon results from a loss of anterior chamber and vitreous volume, causing the anterior chamber and liquified vitreous to leak after the initial incision is made. In all cases of IDS, lifting the iris or preinjection of viscoelastics before commencing the irrigation helps to prevent strong fluctuations of anterior chamber depth, iris-capsular bag diaphragm, and problems which may be caused by it. Patients may experience pain during surgery caused by IDS, which can trigger unexpected abrupt agitation and unwanted movements of patient. This can increase the risk of complications during the surgery. It is therefore very important for surgeons to be aware of the IDS and proper management of it intraoperatively.

In this study, posterior bowing of the iris-lens diaphragm was not observed in nonvitrectomized eyes when irrigation was initiated, and posterior bowing was only slightly evident in the core vitrectomized eyes. However, in the totally vitrectomized eyes, the iris-lens diaphragm was significantly displaced posteriorly, and the anterior chamber was significantly deepened. Eyes that have undergone meticulous vitrectomy are at a higher risk for this syndrome.

IDS can also occur in axial myopia, because of their zonular laxity. The combination of these anatomic factors allow the iris and capsular bag to move posteriorly with the introduction of pressurized fluid into the anterior chamber [6]. To stabilize the anterior chamber, a second instrument was used to lift the iris for a few seconds, allowing fluid to pass from the anterior chamber to the vitreous cavity, resulting in pressure equilibration.

In our study, movement of the irrigation fluid of the anterior chamber into the posterior cavity by lifting the iris worked to stabilize the anterior chamber. This procedure is a safe option for stabilizing the anterior chamber. Once satisfactory anterior chamber depth is achieved, the lens can be removed in the usual manner without difficulty [7]. Another management option is to reduce the height of the infusion bottle and to add a second infusion line to shallow the anterior chamber by decreasing the infusion pressure. Although this may bring anterior chamber depth back to normal, it requires a significant amount of time, an additional incision, and supplementary tubing. Careful determination of bottle height is necessary because improper inflow-outflow management will subject the eye to more chamber volatility and increase the risk of posterior capsule rupture [6].

Using the Miyake-Apple technique, one can observe the lens and adjacent structures after cutting the posterior half of an eye and placing it on a transparent slide [8]. The posterior cavity can be observed by dissecting a part of the posterior chamber or by using an endoscope. However, cutting a part of the posterior cavity can cause a deterioration of the posterior structure as well as the flow of fluid. In addition, an endoscopic camera is not appropriate to study fluid dynamics as it only views a part of the posterior cavity and has low resolution.

Imaging systems such as computed tomography (CT) or magnetic resonance imaging (MRI) can be used to observe fluid flow without disturbing the intact structure. However, it is difficult to observe the dynamic flow of fluid with CT or MRI as these modalities typically only acquire images at a specific moment in time, as opposed to acquiring them over a period of time. While microparticles can be used to study anterior chamber fluid dynamics, they cannot be used in the studies of intraocular fluid dynamics between anterior and posterior chamber.

Diagnostic and interventional radiology have a continuing requirement for dose-efficient X-ray-based imaging techniques to visualize moving anatomic structures, organs, and/or clinical items of interest. Such imaging techniques are commonly associated with contrast medium-aided examinations of the gastrointestinal tract, cardiovascular system, and various other soft tissue organs and structures. One of the advantages of DFS is that it affords the possibility to observe the shape of a structure and the movement of contrast medium while maintaining the intact internal structure [9]. Due to the high resolution of the DFS used in this study, clear images were obtained with diluted contrast medium. Moreover, movement of the iris and lens was observed against the background of the contrast medium, the movement of the contrast medium was identified in real time, and a 30-frame-per-second video clip was successfully obtained.

In this study, we could observe the different changes in anterior chamber depth and the amount of contrast medium that had passed through the posterior cavity in real-time DFS. This is the first time that DFS has been applied in this way in ophthalmology.

The resolution of this imaging technology can be a limitation, although in this study it offered no challenges to identifying the fluid passage and accumulation in the posterior cavity. Moreover, this DFS method can be used to visualize the experimental outcomes of pharmacologic vitreolysis in vivo [5].

Using ultrasound images to observe the vitreous status in vivo, there is an impedance of the crystalline lens on image quality, especially the area directly behind the crystalline lens. But the crystalline lens did not affect the resolution of DFS in the experimental model and afforded the advantage of examining the reaction process in real time [5].

Iodixanol is the only contrast medium formulated with sodium and calcium in a ratio equivalent to that of blood. Iodixanol, a water-soluble contrast medium, is used in coronary angiography with DFS [10]. While iodixanol is well tolerated by the vascular endothelium and has a wide margin of safety, some studies have reported morphologic changes of endothelial cells and vasoreactivity [11–13]. However, to date there have been no studies regarding the toxicity of contrast medium on the human eye. If a study were to be conducted investigating the toxicity of iodixanol on the human eye, the DFS could be applied to study the phacodynamics in the eye.

In conclusion, in this study we could successfully observe real-time images of anterior and posterior cavity using DFS, demonstrating differences in fluid dynamics during phacoemulsification in eyes with or without prior vitrectomy. In the completely vitrectomized eyes, the significantly deep anterior chamber which developed during phacoemulsification could be normalized by allowing the fluid shift to the posterior cavity. Using the digital fluoroscopy system we could successfully identify intraocular fluidics between anterior chamber and posterior cavity. In the group of vitrectomized eyes, a significantly large amount of fluid flowed to the posterior cavity which then led to a normalized anterior chamber depth. Recognizing the differences in the fluidics of the vitrectomized eye as compared to those of the nonvitrectomized eyes may reduce the frequency of intraoperative complications. This study on vitrectomized swine eyes suggests that DFS represents a favorable experimental method to study the intraocular fluid dynamics in vivo.

## Figures and Tables

**Table 1:** Physical properties of iodixanol (Visipaque).

Formula	C_35_H_44_I_6_N_6_O_15_
Molecular mass	1550.191
Concentration of iodine (mgI/mL)	320
Osmolality (mOsmol/kg water)	290
Viscosity (cP)	
20°C	26.6
37°C	11.8
Density (g/mL)	
20°C	1.369
37°C	1.356
Protein binding	Negligible
Metabolism	Excreted unchanged
Half-life	2.1 hours
Excretion	Renal

## References

[1] Szijártó Z., Haszonits B., Biró Z., Kovács B. (2007). Phacoemulsification on previously vitrectomized eyes: results of a 10-year-period. *European Journal of Ophthalmology*.

[2] Ahfat F. G., Yuen C. H. W., Groenewald C. P. (2003). Phacoemulsification and intraocular lens implantation following pars plana vitrectomy: a prospective study. *Eye*.

[3] Zaheer I., Taylor S. R. J., Pearson R. V. (2007). Phacoemulsification in vitrectomized eyes under topical anesthesia. *European Journal of Ophthalmology*.

[4] Yamamoto Y., Uno T., Shisida K. (2006). Demonstration of aqueous streaming through a laser iridotomy window against the corneal endothelium. *Archives of Ophthalmology*.

[5] Bae J. H., Park H. S., Kim J. M. (2015). In vivo assessment of pharmacologic vitreolysis in rabbits with the digital fluoroscopy system. *Investigative Ophthalmology and Visual Science*.

[6] Cionni R. J., Barros M. G., Osher R. H. (2004). Management of lens-iris diaphragm retropulsion syndrome during phacoemulsification. *Journal of Cataract and Refractive Surgery*.

[7] Cheung C. M. G., Hero M. (2005). Stabilization of anterior chamber depth during phacoemulsification cataract surgery in vitrectomized eyes. *Journal of Cataract and Refractive Surgery*.

[8] Pereira F. A. S., Werner L., Milverton E. J., Coroneo M. T. (2009). Miyake-Apple posterior video analysis/photographic technique. *Journal of Cataract and Refractive Surgery*.

[9] Cowen A. R., Davies A. G., Sivananthan M. U. (2008). The design and imaging characteristics of dynamic, solid-state, flat-panel x-ray image detectors for digital fluoroscopy and fluorography. *Clinical Radiology*.

[10] Brunette J., Mongrain R., Rodés-Cabau J., Larose É., Leask R., Bertrand O. F. (2008). Comparative rheology of low- and iso-osmolarity contrast agents at different temperatures. *Catheterization and Cardiovascular Interventions*.

[11] Ramponi S., Grotti A., Morisetti A., Vultaggio S., Lorusso V. (2007). Effects of iodinated contrast media on endothelium: an in vitro study. *Toxicology in Vitro*.

[12] Sendeski M., Patzak A., Pallone T. L., Cao C., Persson A. E., Persson P. B. (2009). Iodixanol, constriction of medullary descending vasa recta, and risk for contrast medium-induced nephropathy. *Radiology*.

[13] Franke R.-P., Fuhrmann R., Hiebl B., Jung F. (2008). Influence of various radiographic contrast media on the buckling of endothelial cells. *Microvascular Research*.

